# Large language models (LLMs) might be the future research language of nucleic acid

**DOI:** 10.1097/JS9.0000000000002694

**Published:** 2025-06-20

**Authors:** Chiranjib Chakraborty, Manojit Bhattacharya, Arpita Das, Md. Aminul Islam

**Affiliations:** a Department of Biotechnology, School of Life Science and Biotechnology, Adamas University, Kolkata, West Bengal, India; b Department of Zoology, Fakir Mohan University, Vyasa Vihar, Balasore, Odisha, India; c COVID-19 Diagnostic Lab, Department of Microbiology, Noakhali Science and Technology University, Noakhali, Bangladesh; d Advanced Molecular Lab, Department of Microbiology, President Abdul Hamid Medical College, Karimganj, Kishoreganj, Bangladesh

*Dear Editor*,

Artificial intelligence (AI) presents immense opportunities for solving the complex problems of different fields, including the biological sciences. Different complex tasks can be performed using the amalgamation of automation and AI, and it can even exceed human performance. That is why the use of AI is increasing steadily. On the other hand, when working with AI, transparency is essential during reporting. In this direction, a recent article published by Agha *et al* illustrated the transparency benchmarks for reporting AI and listed those benchmarks^[1]^. Similarly, Haibe-Kains *et al* discussed the reproducibility and transparency of AI^[2]^. However, both articles highlighted two vital topics: transparent and reproducible AI research. Therefore, both articles are significant and timely.

Large language models (LLMs), a subset of AI, have recently gained massive attention worldwide following the release of ChatGPT on 30 November 2022, in San Francisco, USA. This model is a free online version 3.5 of the GPT-3 (Generative Pretrained Transformer 3) series. The model has emerged as the fastest-growing tool globally. Within 2 months after its launch, the number of users reached about 100 million^[3]^. The model is immensely used in different medical science spheres^[4,5]^. At the same time, it is also utilized in other fields, including drug discovery^[6,7]^, nucleic acid research^[8]^, biomedical engineering^[9]^, and law^[10]^. Due to the excessive use of LLMs, researchers anticipate that research in various fields, ranging from medical science to chemistry to law, will attain a new dimension. LLMs, such as GPT-4, specifically the series 4 model, can generate images and human-like text from any prompt^[11]^. LLMs can solve complex problems related to nucleic acids, and scientists have started exploring the use of LLMs in this area. CodonBERT, an LLM platform, can help to design and optimize mRNA. Recently, mRNA-based vaccines and therapeutics have emerged as highly effective molecules. Therefore, to design and optimize a stable mRNA, sequence optimization is necessary to produce low-cost, high-potency, and safe vaccines and therapeutics. CodonBERT helps develop an optimized mRNA sequence for mRNA-based vaccines and therapeutics^[12]^. Recently, another LLM platform, entitled LitSumm, was developed to help summarize the literature on ncRNAs (non-coding RNAs)^[13]^. Furthermore, Jorapur *et al* stated that LLMs can design primers for diagnostic PCR (polymerase chain reaction)^[14]^. LLMs are capable of answering questions and making accurate decisions. However, LLMs apply neural network architectures to function.

LLMs can take the text and image inputs. Subsequently, it provides the text outputs. It shows human-level performance in the academic arena and various other professional fields. GPT-4 can solve a broader range of problems compared to its predecessor. Therefore, scientists are excited to use the GPT-4 model. This LLM, specifically GPT-4, has created a next-generation platform for researchers known as the multimodal large language model (MLLM). OpenAI’s effort created a new milestone in the Chatbot landscape when it released GPT-4. Following its release, GPT-4 marks the beginning of a new era for AI-powered LLMs. For medical applications, Lee *et al* found that GPT-4 may be a powerful tool, providing valuable responses^[15]^. This powerful GPT-4 model generates marker gene-related material from the typical single-cell RNA-seq study^[16]^. It is an automated cell-type annotation method, which is cost-effective.

The traditional transformer model utilizes attention and self-attention mechanisms, enabling the model to capture information from preceding tokens. The GPT models use a transformer framework. However, these possess a remarkable generative pretraining method. Compared to traditional transformers, which rely on labeled datasets for training, GPTs utilize an enormous corpus of unlabeled data and possess task-specific fine-tuning features^[17]^. Different versions of the GPT models were introduced, and the third generation, i.e., GPT-3, utilizes a remarkably high number of tokens (0.3 trillion) and parameters (0.175 trillion) for training. Due to this magnitude of large numbers, fine-tuning is often not essential in various tasks. The advanced version, recently launched, is the fourth generation, specifically GPT-4. Brynjolfsson *et al* noted in their study that the GPT-4 utilizes around 13 trillion tokens and 1.8 trillion parameters for training^[18]^. A novel feature of GPT-4 compared to GPT-3 is that GPT-4 can generate images from input datasets. Other significant LLMs are Gemini 2.0, developed by Google DeepMind^[19]^, and DeepSeek^[20]^, developed by the Chinese-based company High-Flyer. These LLMs are now GenAI-based, significant analytical models in the domains of biological science and medical research, including nucleic acid analysis.

LLMs are now at the forefront of providing effective solutions in medical science, especially in education, clinical, and research-related solutions^[4,21]^. LLMs can provide solutions in different fields besides the medical domain. White has informed us that LLMs can provide solutions to different areas of chemistry. He described LLMs as potentially the future of chemistry, which will help initiate a new era of developments in the field^[22]^. Recently, Chatterjee *et al* demonstrated that LLMs aid in solving various nucleic acid research problems. They informed us that LLMs will revolutionize nucleic acid research^[8]^. LLMs are capable of transforming different areas of nucleic acid research. Genomic research has also been explored using LLMs. DNABERT, a nucleic acid domain-specific LLM, can aid in evaluating global and transferable genomic DNA sequences, considering the altered environments of upstream and downstream nucleotides. It has the potential to predict genome-wide regulatory elements and reveal their usage frequency^[23]^. Additionally, the DNABERT platform helps identify lncRNA from the assemblies of the plant genome. Using DNABERT, Danilevicz *et al* accurately predicted lncRNAs from genomic sequences, with the highest accuracy noted in *Zea mays* at 83.4%. Similarly, the lowest accuracy was noted in the *Brassica rapa* as 57.9%^[24]^. In another LLM study, Sultan *et al* analyzed the genes associated with cancer predisposition. They used a study involving 53 patients with pathogenic mutations (CPG mutations) and noted that Rb1 was the most commonly mutated gene in the cohort. Other predicted genes were VHL, NF1, and TP53. In this analysis, the predicted genes were 93% correct. However, they noted that LLM shows promise in predicting CPG mutations^[25]^. Recently, Elsborg and Salvatore analyzed biomarkers at the single-cell level to gain a deeper understanding of the disease landscape using LLMs and explainable machine learning (ML). They found the symbolic and straightforward nature of fetched gene signatures. It may enable researchers to elucidate the underlying molecular causes of diseases^[26]^. Similarly, a large-scale pre-trained deep language model tool has been developed to annotate the cell type of single-cell RNA-seq data. This model, named scBERT, can be fine-tuned to utilize supervised user-specific single-cell RNA-seq (scRNA-seq) data. The model validated its performance in terms of robustness to batch effects, novel cell type discovery, and cell type annotation, among others^[27]^. Yamada and Hamada developed a nucleotide language model using tokens similar to those employed in GPT models. Here, tokens are referred to as sequences of words or their particular nucleotide positions inside a sequence. Here, they also consider the k-mers of the sequence, which are the 3-mers and the 4-mer sequences. The following 3-mers were used: TAC, GTA, and CGT. Similarly, the 4-mers were used as GTAC, CGTA, and ACGT. Their nucleotide language model was used to predict RNA–protein interactions^[10]^. Zhang *et al* have developed an RNA language model that performs multiple sequence alignment, which is an unsupervised RNA-MSM (Multiple Sequence Alignment-Associated RNA Language Model). It can map direct base pairing information and also provide solvent accessibility knowledge^[28]^. Another significant problem in this research domain. Recently, Song *et al* developed GLMSite, which accurately identifies DNA- and RNA-binding sites using a geometric graph learning (GGL) model^[29]^. GGL is an ML-based method that uses graphs to analyze and represent data. On the other hand, protein language models (pLMs), a type of LLM, are among the significant deep learning (DL)-based models that utilize natural language processing (NLP) techniques to analyze and comprehend protein sequences. Similarly, using pLMs, Roche *et al* developed the EquiPNAS model for improved prediction of protein-nucleic acid binding sites. The model combines the strengths of symmetry-aware deep graph learning and pre-trained language models (PLMs)^[30]^. Therefore, nucleic acid research is progressing quickly to solve the diverse problems in this area. Recently, different DNA language models have been developed to comprehend the sequence context in the human genome. In this direction, a DNA language model, GROVER, has been developed to learn the context of the human genome sequence using next-k-mer prediction with Byte-Pair Encoding (BPE) and a tokenization-based model architecture. This model illustrates the DNA’s coding for proteins and transcripts^[31]^.

Recently, scientists have utilized LLMs to develop nucleic acid language models (NALMs), which can be trained using nucleic acid sequences, such as RNA or DNA. The NALMs are capable of comprehending and predicting the behavior of these biological molecules. In these NALMs, sequences are described as text and employ tokenization to split them down into smaller units, known as tokens. NALM is also referred to as a genomic language model (gLM)^[32]^.

Just like LLMs, NALMs aim to comprehend the meaning of sequences in terms of their structure and function. It will offer new possibilities for investigating problems in biological science and developing solutions to them, thereby bridging the gap between medical science and biological science^[32]^.

Different scientists are attempting to understand various aspects of nucleic acids through LLMs, NALMs, or gLMs. Most importantly, we understand the various crucial aspects of nucleic acids, including evaluation metrics, species specificity, nucleotide sequence interpretation, and the complexities of LLMs. However, the proper tokenization process in converting to DNA/RNA sequences is essential.

The state-of-the-art LLMs, such as GPT-4, Google DeepMind, and DeepSeek, have emerged as leaders in AI innovation in nucleic acid research. Conversely, several LLM-derived models are assisting in solving the critical problem of nucleic acid research by handling a massive amount of data in this domain. Overall, LLMs can potentially revolutionize nucleic acid research by training on large datasets that incorporate texts, images, and videos. It indicates that it will open up a promising avenue for nucleic acid research. However, the potential replacement of humans and pressing ethical issues are considerable concerns. At the same time, progress in scientific development must continue. In the future, LLMs are expected to continue improving and will be introduced as next-generation, upgraded models. The future research language of nucleic acids may depend on MLLMs. Therefore, it is time for researchers to rethink nucleic acid-related research experiments, tools, and experimental design. They should utilize advanced next-generation technologies and tools, such as LLM or MLLM, to make rapid progress in nucleic acid research.

## Data Availability

The authors confirm that the data supporting the findings of this study are available within the article.
